# Prevalence of Menstrual Irregularities in Psychiatric Patients and Its Impact on Treatment Adherence: A Cross-Sectional Study

**DOI:** 10.1192/bjo.2025.10183

**Published:** 2025-06-20

**Authors:** Pooja Shakya, Poulami Basu, Rupam Kumari, Manoj Kumar

**Affiliations:** ^1^Institute of Human Behaviour and Allied Sciences, Delhi, India; ^2^All India Institute of Medical Sciences, Delhi, India

## Abstract

**Aims:** Menstrual irregularities (MI) are a frequently overlooked yet clinically significant concern among women with psychiatric disorders. Several psychiatric conditions, particularly schizophrenia and bipolar disorder, involve dopaminergic dysregulation, which may contribute to hormonal disturbances. Antipsychotic medications, especially typical antipsychotics, are known to impact menstrual cycles through their effects on dopamine pathways, leading to hyperprolactinemia and subsequent menstrual dysfunction. However, research on the prevalence of MI and its relationship with psychiatric illness and treatment adherence remains limited, particularly in the Indian context. This study aims to assess the prevalence of MI among female psychiatric inpatients and explore its association with socio-clinical factors, antipsychotic medication use, and treatment adherence.

**Methods:** A cross-sectional study was conducted at a tertiary care mental health institute, recruiting 100 female inpatients diagnosed with psychiatric disorders. MI was defined as any deviation from a regular menstrual cycle, including oligomenorrhea, amenorrhea, or irregular bleeding. Menstrual distress was assessed using the MEDI-Q (Menstrual Distress Questionnaire) scale, while treatment adherence was evaluated with the Brief Adherence Rating Scale (BARS). Statistical analysis examined associations between MI, antipsychotic use, prolactin levels, psychiatric diagnosis, and treatment adherence.

**Results:** The mean age of participants was 36.5 years, with an average illness duration of 3.6 years. Psychiatric diagnoses included psychotic disorders (62%), bipolar disorder (22%), depressive disorder (10%), and neurotic disorders (6%). Antipsychotic medication use was recorded in 82% of participants. The overall prevalence of MI was 37%. Among patients with psychotic disorders, 50% exhibited MI, with a significantly higher prevalence in those on typical antipsychotics (80.7%) compared with atypical antipsychotics (27.7%). MI was also observed in 31.5% of bipolar patients on atypical antipsychotics. Patients with poor treatment adherence (<50% on BARS – Brief Adherence Rating Scale) showed significantly higher score for MEDI-Q Total Score (16.51 ± 12.99 vs. 10.86 ± 12.36; p < 0.01) as well as for the subscales MSD (Menstrual Symptom Distress) and MESI (Menstrual Specificity Index). The menstrual distress was associated to being on antipsychotics; in fact, MEDI-Q Total Score was significantly higher in women on antipsychotics as compared with those not on antipsychotics.

**Conclusion:** Menstrual irregularities are prevalent among female psychiatric inpatients, particularly those with psychotic disorders and those on typical antipsychotics. These disturbances negatively impact medication adherence, highlighting the need for routine menstrual health assessments, prolactin monitoring, and personalized treatment approaches to balance psychiatric stability with reproductive health. Addressing patient concerns regarding menstrual side effects may improve adherence and overall treatment outcomes.

